# Associations of hypertension burden on subsequent dementia: a population-based cohort study

**DOI:** 10.1038/s41598-021-91923-8

**Published:** 2021-06-10

**Authors:** Hyunjean Jung, Pil-Sung Yang, Daehoon Kim, Eunsun Jang, Hee Tae Yu, Tae-Hoon Kim, Jung-Hoon Sung, Hui-Nam Pak, Moon-Hyoung Lee, Gregory Y. H. Lip, Boyoung Joung

**Affiliations:** 1 Division of Cardiology, Department of Internal Medicine, Severance Cardiovascular Hospital, Yonsei University College of Medicine, 50-1 Yonsei-ro, Seodaemun-gu, Seoul, 03722 Republic of Korea; 2 Department of Cardiology, CHA Bundang Medical Center, CHA University, Seongnam, Republic of Korea; 3 Liverpool Centre for Cardiovascular Science, University of Liverpool and Liverpool Heart & Chest Hospital, Liverpool, England UK

**Keywords:** Hypertension, Dementia

## Abstract

In this nationwide cohort study, we assessed the effects of hypertension burden and blood pressure (BP) control on dementia in different age subgroups. From the Korean National Health Insurance Service-Health Screening cohort from January 1, 2005 to December 31, 2013, we enrolled 428,976 subjects aged 40–79 years without previous diagnosis of dementia or stroke. During a mean follow-up of 7.3 ± 1.5 years, 9435 (2.2%) were diagnosed with dementia. Per 10 mmHg increase in systolic BP (SBP), risk of dementia was increased by 22% (95% confidence interval [CI] 1.15–1.30) in subjects aged 40–59 years and 8% (95% CI 1.04–1.11) in subjects aged 60–69 years. No significant associations were observed in subjects aged ≥ 70 years. Among subjects aged 40–59 years, both vascular and Alzheimer’s dementia risks were increased with increasing SBP. Increasing hypertension burden (proportion of days with increased BP) was associated with higher dementia risk (hazard ratio [HR] 1.09 per 10% increase, 95% CI 1.08–1.10). Among patients with baseline SBP ≥ 140 mmHg, optimal follow-up SBP (120–139 mmHg) was associated with decreased dementia risk (HR 0.69, 95% CI 0.50–0.95). Hypertension burden was associated with higher risks of dementia. Adequate BP control was associated with lower risk of dementia in individuals aged < 70 years.

## Introduction

Dementia is characterized by a decline in memory, language, problem-solving and other cognitive skills that affects a person's ability to perform everyday activities^1^. Approximately 40 million people worldwide have dementia, and this number is expected to increase up to 115 million by 2050 due to the aging population^2^. To date, no proven interventions are available to prevent or delay dementia.

Hypertension, which affects more than 75% of people > 65 years of age, has emerged as a potential modifiable risk factor for mild cognitive impairment and dementia in observational studies^3^. The relationship between blood pressure (BP) and dementia is well established^4^. Persistent midlife hypertension is associated with increased risk of a late life dementia^5,6^, leading it to be included as a putative risk factor in dementia prevention guidelines^4,7^. However, in several randomized clinical trials (RCTs) about the effects of BP reductions on cognitive outcomes, including a recently published SPRINT MIND, results have generally been inconclusive^8–11^. In systematic reviews, the association of hypertension with cognitive dysfunction and dementia has been suggested to differ based on age and follow-up duration and to contribute to lack of consensus. Therefore, to fully evaluate the association of hypertension and cognitive outcomes, evaluation of the age-specific effects of BP with a sufficiently long follow-up period is needed.

In the present nationwide, population-based study, we investigated the associations of systolic (SBP) and diastolic BP (DBP) level and hypertension burden with dementia risk in wide range of age subgroups. Moreover, we investigated whether BP control over time was associated with reduction of dementia risk.

## Methods

This study was based on the Korean National Health Insurance Service-Health Screening (NHIS-HealS) cohort released in 2015^12,13^. The general health screening program can be applied at least once every 2 years for the entire population of Korean adults aged 40 years or older^14^. The cohort consisted of 514,764 Koreans as an initial 2002 cohort and followed subjects through 2013 on data including sociodemographic information, diagnoses based on the 10th revision of the International Classification of Disease-10 (ICD-10) codes, admission, treatment, and National Health Screening data^12,13^. National Health Screening is conducted biennially and includes regular blood tests, chest X-rays, physical examinations, and medical history questionnaires. Information on death (date and cause) from Statistics Korea was individually linked using unique personal identification numbers^12,13^. This study was approved by the Institutional Review Board of Yonsei University Health System (4-2014-0996), and informed consent was waived.

### Study population

**Figure 1:**
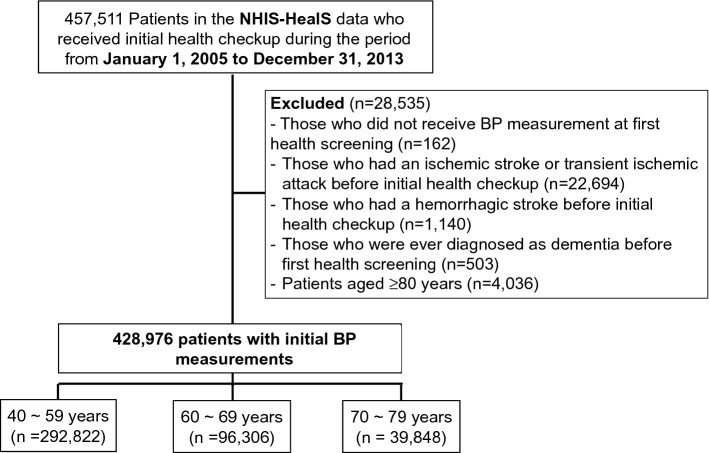
Flowchart of the study population. *BP* blood pressure, *NHIS-HealS* National Health Insurance Service-Health Screening cohort.

The first three years of the cohort (2002 ~ 2004) were set as the window period for obtaining baseline comorbidities and drug use of the study population. Therefore, 57,253 subjects who had undergone initial National Health screening between January 2002 to December 2004 were excluded from the initial study population. Subsequently, adults ≥ 40 years of age who underwent initial National Health screening between January 1, 2005 and December 31, 2013 (n = 457,511) were identified from the NHIS-HealS cohort^13^. Subjects with the following were excluded: (i) BP not measured at initial health screening (n = 162); (ii) history of ischemic stroke or transient ischemic attack before initial health screening (n = 22694); (iii) history of hemorrhagic stroke before initial health screening (n = 1140); (iv) previous diagnosis of dementia at index date (n = 503); and (v) age ≥ 80 years (n = 4036). Finally, this study included 428,976 subjects (Fig. 1). The study index date was that of initial health screening. Prespecified age subgroups were as follows: 40–59, 60–69, and 70–79 years.

### BP measurement

BP measurements were performed at local hospitals and clinics certified as medical health examination centers by the Korean National Health Insurance Corporation^15–17^. After ≥ 5 min of rest in the sitting position, BP was measured at the brachial artery by qualified medical personnel. Automatic oscillometric devices or mercury sphygmomanometers were used, with the choice of device left to the discretion of individual examination centers. If the first BP measurement was > 120/80 mmHg, the measurement was repeated once to prevent overestimation^16,17^. The main analysis used BP measured at the index date (initial health screening).

### Comorbidities and outcomes

Baseline comorbidities were defined using the medical claims and prescription medication prior to the index date. To ensure diagnostic accuracy, the participants were considered to have comorbidities when the condition was diagnosed at discharge or was confirmed at least twice in an outpatient setting, similar to previous studies using the National Health Insurance Service (NHIS) database^13,16–18^. The detailed definitions of comorbidities are presented in Supplementary Table 1.

The study endpoint was first occurrence of dementia, which was defined as dementia diagnosis combined with use of one or more of the following dementia drugs: rivastigmine, galantamine, memantine, or donepezil. Participants were followed from the index date and censored at occurrence of dementia, death, or December 31, 2013, whichever occurred first.

Use of prescription medication was ascertained by identifying database claims within 90 days before the index date. Income status was evaluated based on total amount of national health insurance premiums paid by the insured individual in the index year, which is proportional to the individual’s income.

The definitions of overall dementia, Alzheimer’s or vascular dementia were based on the recording of relevant ICD-10 codes (F00 or G30 for Alzheimer’s disease; F01 for vascular dementia; and F02, F03, or G31 for other dementia) and the prescription of medication for dementia (rivastigmine, galantamine, memantine, or donepezil). When both codes for Alzheimer’s disease and vascular dementia were recorded, we followed the principal diagnosis. If both were in the additional diagnosis up to the next insurance claim in the database, the subject was classified as other dementia.

To evaluate the accuracy of our definition of dementia, a validation study was conducted in two teaching hospitals with a total of 972 patients who were given the ICD-10 codes F00, G30, F01, F02, and F03. Patient medical records and results of cognitive function tests including the Mini Mental State Examination of these patients were reviewed by three physicians. The positive predictive value was 94.7% (920/972)^19^.

### Statistical analysis

Cox regression with separate models was used for 40–59, 60–69, and 70–79 year age subgroups to estimate the adjusted hazard ratio (HR) for the association between SBP level and dementia. To control for confounding factors, variables included age, sex, income, body mass index, alcohol consumption, smoking, physical activity, hypothyroidism, hyperthyroidism, major bleeding, atrial fibrillation, heart failure, diabetes mellitus, dyslipidemia, myocardial infarction, peripheral artery disease, chronic kidney disease, COPD, liver disease, malignant neoplasm, and medication at baseline (e.g. aspirin, statin, oral anticoagulant, ß-blocker, RAAS blocker, calcium channel blocker, diuretics and alpha blocker) were adjusted in the multivariable models. First, we assessed the effects of SBP measured at baseline on the risk of incident dementia. Restricted cubic spline curves were constructed to examine the effects of continuous values of BP. Four knots were placed at the 5th, 35th, 65th, and 95th percentiles of BP. In addition, categorical variables of BP were used to assess associations with dementia. Sensitivity analysis was performed by censoring individuals at the date of incident ischemic or hemorrhagic stroke during follow-up because stroke could alter the risk of dementia and affect the association between BP and incident dementia.

**Figure 2:**
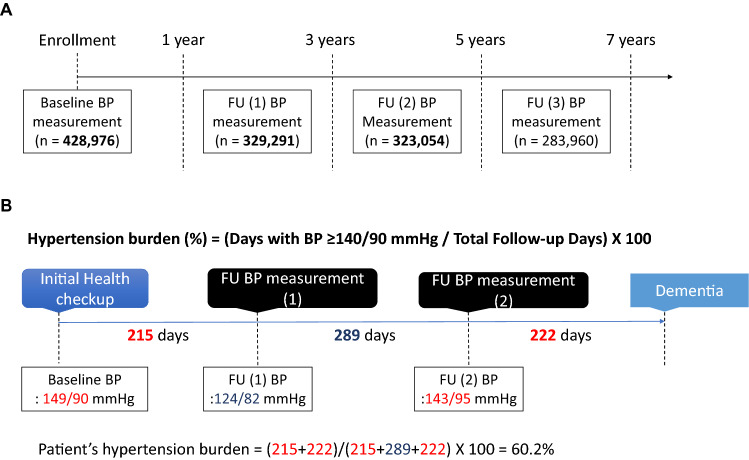
(**A**) Timeline of blood pressure measurements and (**B**) estimation of hypertension burden during follow-up. *BP* blood pressure, *FU* follow-up.

During the follow-up period, BP was updated up to four times at 1–3 years interval (Fig. 2). To evaluate whether the increase or decrease of SBP over time could affect future risk of dementia, change of SBP was calculated as the difference between baseline and follow-up SBP (i.e., delta SBP). Follow-up SBP was defined as the earliest measured SBP that was measured 1–3 years after the initial BP measurement. To investigate the cumulative effects of hypertension on dementia risk, we assessed the association of each patient’s hypertension burden during follow-up with subsequent dementia risk. In this study, hypertension burden was defined as the proportion of days with increased BP (at least 140/90 mmHg) to the observation time interval, based on baseline and follow-up BP measurements (Fig. 2).

Descriptive statistics were used to characterize baseline characteristics and comorbidities. Continuous variables were expressed as the mean ± standard deviation (SD), and categorical variables were reported as frequency (percentage). Baseline characteristics were compared across the three groups using the Chi-square test or Fisher’s exact test for categorical variables and the ANOVA or Kruskal–Wallis test for continuous variables. Incidence rates of dementia were calculated by dividing the number of events by person-time at risk, with the 95% confidence interval (CI) estimated by exact Poisson distribution. Exact test was used in comparing two incidence rates. All tests were two-tailed, with P-value < 0.05 considered statistically significant. Statistical analyses were conducted using SAS version 9.3 (SAS Institute, Cary, NC, USA), SPSS version 23.0 statistical package (SPSS Inc., Chicago, IL, USA), and R statistical software, version 3.5.3 (R Foundation for Statistical Computing, Vienna, Austria).

## Results

During the 2005–2013 period, 428,976 participants aged 40–79 years with no previous diagnosis of dementia underwent health screenings including BP measurements; 54.4% were male, and the mean age was 55.5 ± 8.9 years. The proportions of age subgroups 40–59 years, 60–69 years, and 70–79 years were 68.3% (n = 292,822), 22.5% (n = 96,306), and 9.3% (n = 39,848), respectively. BP level and the proportion of comorbidities including hypertension, hyperthyroidism and atrial fibrillation were highest in population aged 70–79 years (Supplementary Table 2).

During the mean follow-up duration of 7.3 ± 1.5 years, 9435 dementia events were observed. The crude incidence rates for dementia were 0.05 (95% confidence interval, CI 0.04–0.05), 0.51 (95% CI 0.50–0.53), 1.88 (95% CI 1.83–1.93) per 100 person-years in subjects 40–59, 60–69, and 70–79 years of age, respectively. The mean time to develop dementia was 7.4 ± 1.3, 7.1 ± 1.6 and 6.5 ± 2.0 years in age 40–59, 60–69 and 70–79 years, respectively. Dementia according to the time in different BP group (Supplementary Fig. 1), the number of demented patients per age group and types of dementia diagnosed (Supplementary Table 3) and distribution of SBP and DBP (Supplementary Fig. 2) are presented in online materials. Baseline characteristics based on dementia status at the end of follow-up are presented in Table 1. Patients who developed dementia were older, less active, and had lower income and more comorbidities including heart failure and diabetes than subjects who did not develop dementia.

**Table 1:** Baseline characteristics of patients based on dementia status at the end of follow-up.

Variables	Overall (n = 428,976)	No dementia (n = 419,541)	Dementia (n = 9,435)	p value
Age, years	55.5 ± 8.9	55.2 ± 8.7	68.4 ± 7.0	< 0.001
Male	233,380 (54.4)	229,557 (54.7)	3823 (40.5)	< 0.001
Systolic blood pressure, mmHg	125.8 ± 16.6	125.7 ± 16.6	131.1 ± 18.4	< 0.001
Diastolic blood pressure, mmHg	78.4 ± 10.7	78.4 ± 10.7	79.3 ± 11.0	< 0.001
Hypertension diagnosis at baseline	126,525 (29.0)	121,369 (29.0)	5156 (55.0)	< 0.001
Hypertension duration at baseline, months	3.1 ± 1.7	3.1 ± 1.7	3.2 ± 1.6	0.001
High tertile of income	237,043 (54.7)	232,311 (55.4)	4732 (50.2)	< 0.001
Body mass index, kg/m^2^	24.0 ± 2.9	24.0 ± 2.9	23.6 ± 3.1	< 0.001
**Alcohol consumption, times/week**				< 0.001
< 1	288,206 (73.3)	280,998 (73.1)	7208 (81.9)	
1–2	64,669 (16.4)	63,952 (16.6)	717 (8.1)	
3–4	25,523 (6.5)	25,131 (6.5)	392 (4.5)	
≥ 5	15,030 (3.8)	14,545 (3.8)	485 (5.5)	
Missing values	35,548	34,195	633	
**Smoking**				< 0.001
Never	290,814 (71.6)	283,677 (71.4)	7137 (80.2)	
Ex	37,707 (9.3)	37,126 (9.3)	581 (6.5)	
Current	77,524 (19.1)	76,348 (19.2)	1176 (13.2)	
Missing values	22,931	22,390	541	
**Physical activity, times/week**				< 0.001
0–2	300,056 (76.8)	293,085 (76.8)	6971 (80.1)	
3–4	48,643 (12.5)	48,047 (12.6)	596 (6.8)	
≥ 5	41,842 (10.7)	40,707 (10.7)	1135 (13.0)	
Missing values	38,435	37,702	733	
Hypothyroidism	10,739 (2.5)	10,452 (2.5)	287 (3.0)	< 0.001
Hyperthyroidism	10,670 (2.5)	10,354 (2.5)	316 (3.3)	< 0.001
Major bleeding	4017 (0.9)	3838 (0.9)	179 (1.9)	< 0.001
Atrial fibrillation	3754 (0.9)	3552 (0.8)	202 (2.1)	< 0.001
Heart failure	12,300 (2.9)	11,438 (2.7)	862 (9.1)	< 0.001
Diabetes mellitus	33,025 (7.7)	31,394 (7.5)	1631 (17.3)	< 0.001
Dyslipidemia	100,786 (23.5)	97,654 (23.3)	3132 (33.2)	< 0.001
Myocardial infarction	4043 (0.9)	3845 (0.9)	198 (2.1)	< 0.001
Peripheral artery disease	7672 (1.8)	7357 (1.8)	315 (3.3)	< 0.001
Chronic kidney disease	2957 (0.7)	2847 (0.7)	110 (1.2)	< 0.001
COPD	39,440 (9.2)	37,321 (8.9)	2119 (22.5)	< 0.001
Liver disease	88,432 (20.6)	85,962 (20.5)	2470 (26.2)	< 0.001
Malignant neoplasm	28,304 (6.6)	27,269 (6.5)	1035 (11.0)	< 0.001
**Medication at baseline**				
Aspirin	49,560 (11.6)	47,406 (11.3)	2154 (22.8)	< 0.001
Statin	40,145 (9.4)	38,831 (9.3)	1314 (13.9)	< 0.001
Oral anticoagulant	1255 (0.3)	1190 (0.3)	65 (0.7)	< 0.001
ß-blocker	50,899 (11.9)	48,761 (11.6)	2138 (22.7)	< 0.001
RAAS blocker	52,025 (12.1)	49,927 (11.9)	2098 (22.2)	< 0.001
Calcium channel blocker	66,124 (15.4)	63,389 (15.1)	2735 (29.0)	< 0.001
Diuretics	60,560 (14.1)	57,741 (13.8)	2819 (29.9)	< 0.001
Alpha blocker	14,808 (3.5)	14,180 (3.4)	628 (6.7)	< 0.001

Values are presented as mean ± standard deviation or n (%).

*COPD* chronic obstructive pulmonary disease, *RAAS* renin–angiotensin–aldosterone system.

### SBP, DBP and incidence of dementia

**Figure 3:**
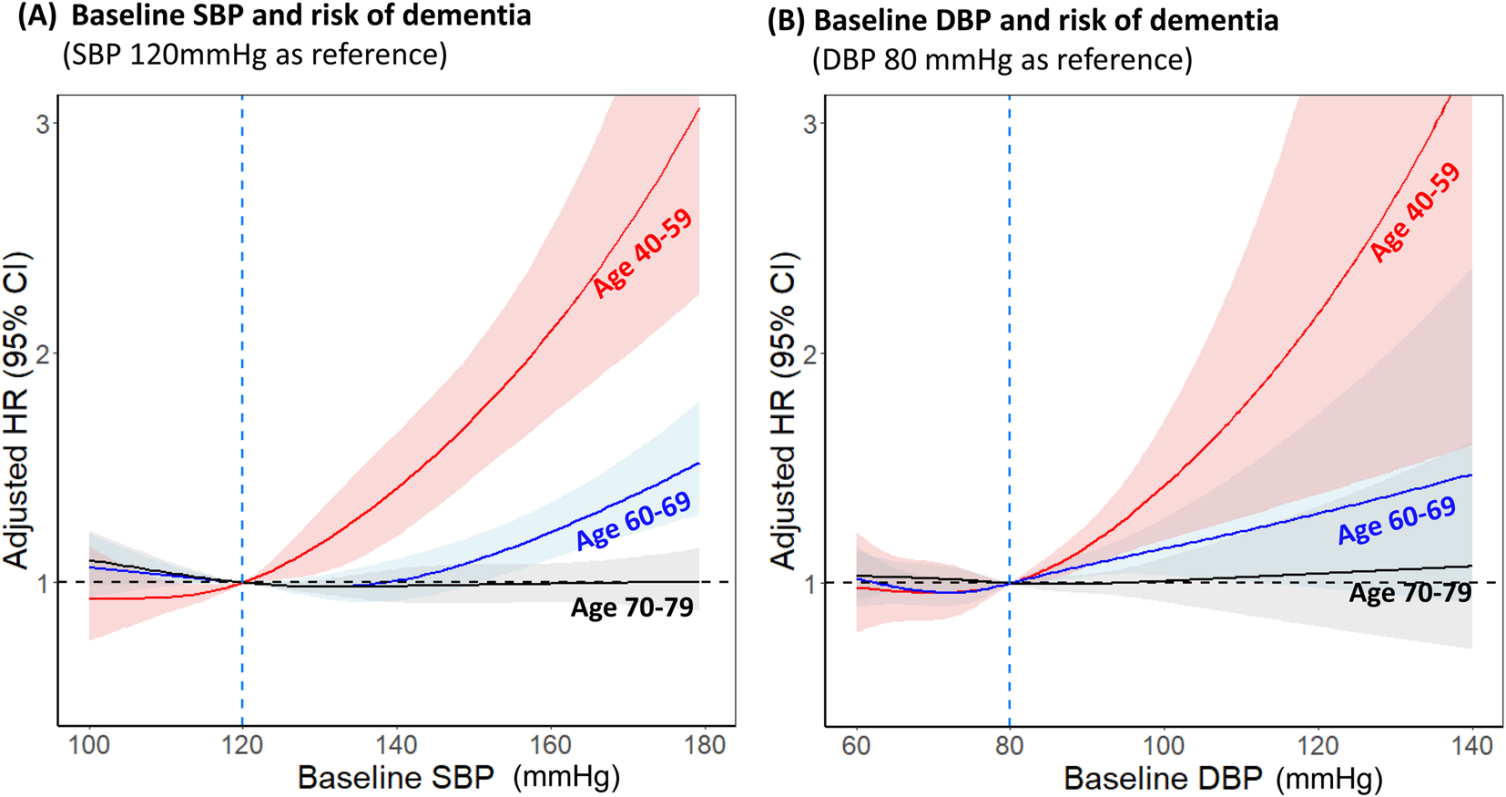
Adjusted HRs for risk of dementia according to baseline (**A**) SBP and (**B**) DBP measured in different age subgroups. Spline curves represent HRs adjusted for age, sex, income, body mass index, alcohol consumption, smoking, physical activity, hypothyroidism, hyperthyroidism, major bleeding, atrial fibrillation, heart failure, diabetes mellitus, dyslipidemia, myocardial infarction, peripheral artery disease, chronic kidney disease, chronic obstructive pulmonary disease, liver disease, malignant neoplasm, and medication at baseline (e.g. aspirin, statin, oral anticoagulant, ß-blocker, RAAS blocker, calcium channel blocker, diuretics and alpha blocker). Subjects with baseline SBP 120 mmHg or DBP 80 mmHg were used as reference. *CI* confidence interval, *DBP* diastolic blood pressure, *HR* hazard ratio, *SBP* systolic blood pressure.

Among individuals with baseline SBP > 120 mmHg, continuous measures of SBP using cubic splines indicated increased risk of dementia with higher SBP in subjects 40–59 years of age (HR per 10 mmHg increase 1.22, 95% CI 1.15–1.30) and in subjects 60–69 years of age (HR per 10 mmHg increase 1.08, 95% CI 1.04–1.11; Fig. 3A). Significant association was not observed with SBP in subjects 70–79 years of age (HR per 10 mmHg increase 0.99, 95% CI 0.97–1.02). In subjects with baseline DBP > 80 mmHg, continuous measures of DBP using cubic splines indicated an increased risk of dementia with higher DBP in subjects 40–59 years of age (HR per 10 mmHg increase 1.20, 95% CI 1.10–1.30) and in subjects 60–69 years of age (HR per 10 mmHg increase 1.07, 95% CI 1.03–1.11). Conversely, significant association was not observed with DBP in subjects 70–79 years of age (Fig. 3B). Similar trend was observed in subjects including previous history of stroke (Supplementary Fig. 3).

Even after censoring for ischemic stroke and hemorrhagic stroke, increased SBP was consistently associated with increased risk of dementia in subjects 40–69 years of age but not in subjects > 70 years of age (Supplementary Fig. 4). In analyses using SBP categories (< 120, 120–139 [reference], ≥ 140 mmHg), SBP ≥ 140 mmHg in subjects 40–59 and 60–69 years of age was consistently associated with increased risk of dementia, with adjusted HRs of 1.76 (95% CI 1.49–2.07) and 1.11 (95% CI 1.02–1.20), respectively (Supplementary Fig. 5).

In analyses using DBP categories (< 80 [reference], 80–90, ≥ 90 mmHg), DBP ≥ 90 mmHg in subjects 40–59 and 60–69 years of age was consistently associated with increased risk of dementia, with adjusted HRs of 1.43 (95% CI 1.20–1.71) and 1.16 (95% CI 1.05–1.27), respectively. (Supplementary Fig. 6).

### BP and incidence of vascular and Alzheimer’s dementia

**Figure 4:**
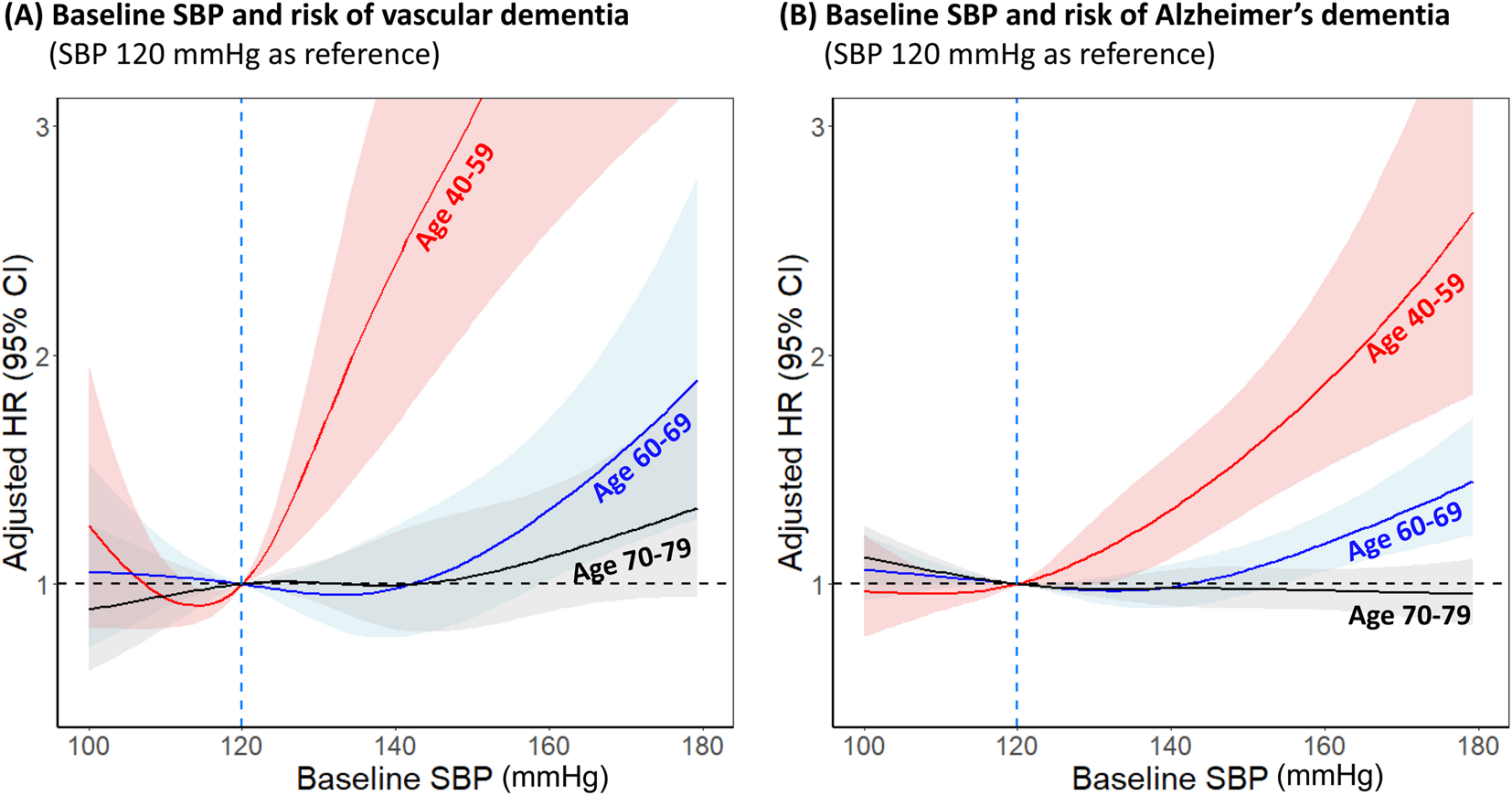
Adjusted HRs for risk of (**A**) vascular and (**B**) Alzheimer’s dementia according to baseline SBP measured in different age subgroups. Subjects with baseline SBP 120 mmHg were used as reference. Spline curves represent HRs adjusted same as Fig. 3. Abbreviations as in Fig. 3.

The risks of vascular and Alzheimer’s dementia associated with increased SBP in different age groups are shown in Fig. 4. In patients with baseline SBP > 120 mmHg, increasing SBP was associated with higher risk of vascular dementia in subjects 40–59 years of age (HR per 10 mmHg increase 1.31, 95% CI 1.18–1.45) and in subjects 60–69 years of age (HR per 10 mmHg increase 1.16, 95% CI 1.07–1.26; Fig. 4A). Increasing SBP was also associated with higher risk of Alzheimer’s dementia in subjects 50–59 years of age (HR per 10 mmHg increase 1.20, 95% CI 1.10–1.30) and in subjects 60–69 years of age (HR per 10 mmHg increase 1.07, 95% CI 1.03–1.11; Fig. 4B). Significant associations were not observed between SBP and the two types of dementia in subjects 70–79 years of age.

### Hypertension burden and dementia risk

**Figure 5:**
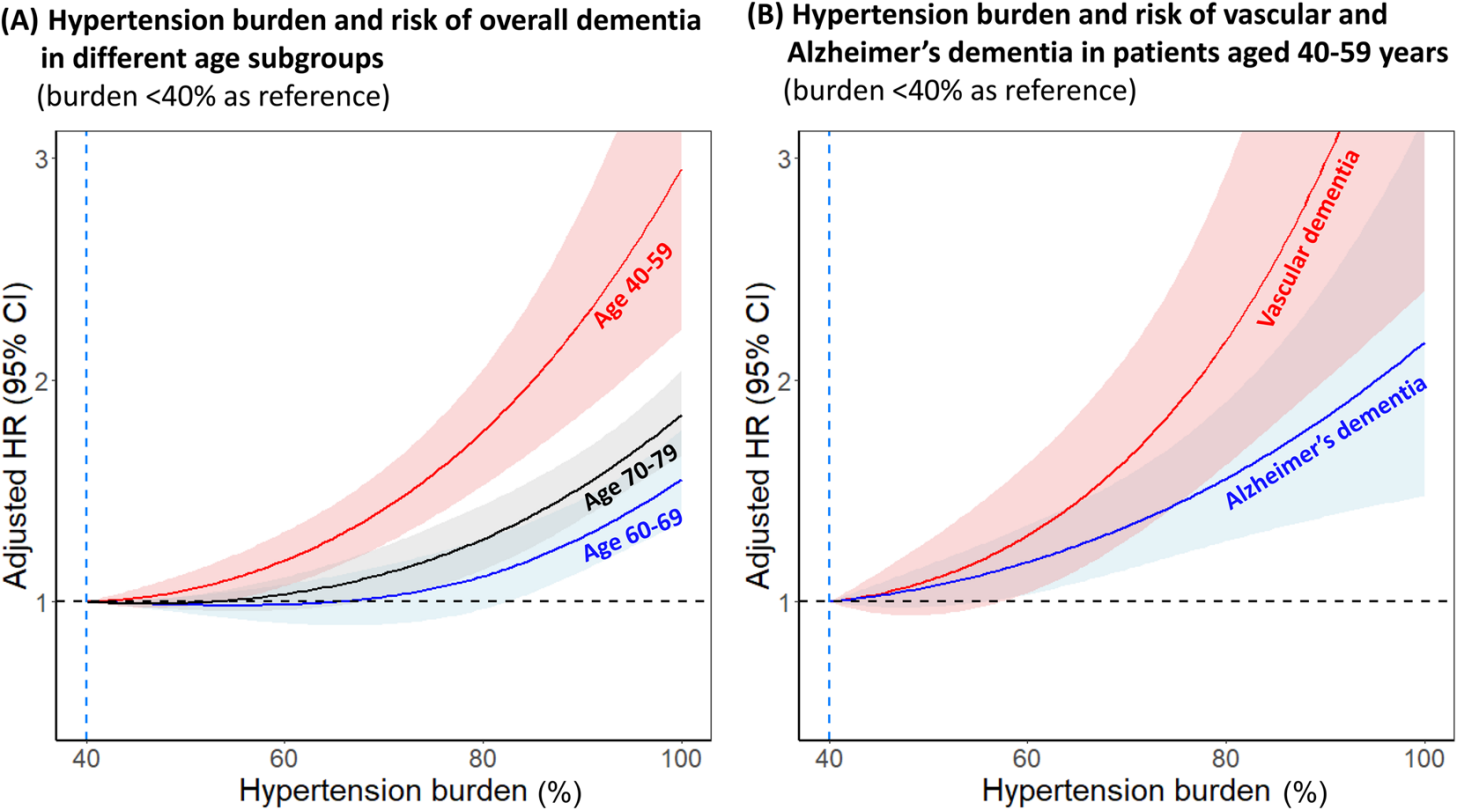
(**A**) Hypertension burden and risk of overall dementia measured in different age subgroups (burden < 40% as reference) (**B**) Hypertension burden and risk of vascular and Alzheimer’s dementia in patients aged 40–59 years (burden < 40% as reference). Spline curves represent HRs adjusted same as Fig. 3. *CI* confidence interval, *HR* hazard ratio.

Figure 5 depicts the risk of dementia associated with continuous measures of hypertension burden during follow-up using restricted cubic spline analyses (burden of < 40% as reference). The cubic spline suggested a log-linear relationship between hypertension burden and the risk of dementia in those aged 40–59 years (Fig. 5A). Increase in hypertension duration continuously increased the adjusted risk of dementia (adjusted HR per 10% increase 1.09, 95% CI 1.08–1.10) and reached an adjusted HR of 2.95 (95% CI 2.23–3.91) at 100% (< 40% as reference). In patients aged 40–59 years, the association of hypertension burden was consistent with the risks of both dementia subtypes (Fig. 5B), more prominently for vascular dementia (adjusted HR 1.11, 95% CI 1.09–1.13) than for Alzheimer Dementia (adjusted HR 1.07, 95% CI 1.06–1.08).

### BP control and incidence of dementia

**Figure 6:**
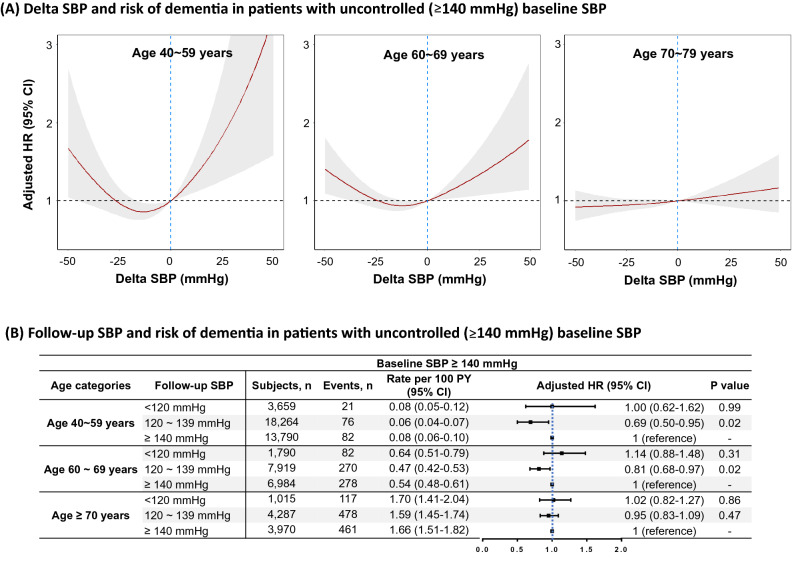
(**A**) Adjusted HR for risk of dementia according to delta SBP in patients with uncontrolled (≥ 140 mmHg) baseline SBP. Delta SBP was defined as follow-up SBP minus baseline SBP. Subjects with delta SBP = 0 mmHg were used as reference. (**B**) Event rates and adjusted HR for risk of dementia according to follow-up SBP in patients with uncontrolled (≥ 140 mmHg) baseline SBP. Rates are per 100 person-years. Subjects with continuously uncontrolled hypertension (follow-up SBP ≥ 140 mmHg) were used as reference. Spline curves represent HRs adjusted same as Fig. 3. Abbreviations as in Fig. 3.

Among 83,868 subjects with baseline SBP ≥ 140 mmHg, 61,678 had follow-up BP measurements. The large positive and negative change of SBP increased the risk of all-cause dementia, showing a U-shaped relationship (Fig. 6A). In subjects 40–59 years, with delta SBP decreasing below baseline to − 25 mmHg, the spline curve indicated a decrease in risk of dementia using subjects with delta SBP = 0 mmHg as reference (nadir point at − 13 mmHg; HR 0.86, 95% CI 0.75–0.97). The risk of dementia increased as delta SBP increased from nadir point (HR per 10 mmHg increase 1.21, 95% CI 1.03–1.44). In subjects 60–69 years, the risk of dementia increased as delta SBP increased over nadir point (HR per 10 mmHg increase 1.11, 95% CI 1.00–1.22). However, a non-significant association between delta SBP and dementia risk was observed among subjects ≥ 70 years of age.

The risk of dementia associated with follow-up SBP in patients with uncontrolled (≥ 140 mmHg) baseline SBP is shown in Fig. 6B. In patients with baseline SBP ≥ 140 mmHg, follow-up SBP 120–139 mmHg was associated with decreased risk of dementia compared with follow-up SBP ≥ 140 mmHg (continuously uncontrolled) in both subjects 40–59 years of age (HR 0.69, 95% CI 0.50–0.95) and 60–69 years of age (HR 0.81, 95% CI 0.68–0.97). No significant associations between follow up SBP and dementia risk was observed among subjects ≥ 70 years of age.

An analysis of effects of different classes of antihypertensive medications on subsequent dementia risk is presented in Supplementary Fig. 7. There is no significant effect on the risk of dementia according to the type of BP medication.

## Discussion

The present study results revealed four principal findings: (i) increased SBP and DBP were associated with increased dementia risk in individuals < 70 years of age but not in subjects > 70 years of age, even after censoring for stroke; (ii) increased hypertension burden was associated with increased risk of dementia, and this association was more pronounced for the risk of vascular disease; (iii) large positive and negative changes of SBP increased the risk of dementia in a U-shaped relationship and optimal follow-up SBP of 120–139 mmHg was associated with reduced dementia risk in subjects < 70 years of age. These findings provide important information regarding the effects of hypertension burden and optimal BP control, which has previously shown significant benefit for cardiovascular morbidity and mortality, on development of dementia based on age.

Current literature is unclear regarding the association between hypertension and cognitive decline or dementia^11^. Systematic reviews have indicated that variations in age and follow-up duration contribute to the lack of consensus^3,20^. In the present study, hypertension (baseline SBP ≥ 140 mmHg or baseline DBP ≥ 90 mmHg) was associated with increased risk of dementia in the general population < 70 years of age but not ≥ 70 years of age, which is consistent with results in numerous studies that showed midlife hypertension to be associated with increased dementia risk in later life^5,6^.

Increased dementia risk associated with high BP was present in subjects free of stroke at baseline, even after censoring for future stroke. This suggests that subclinical vascular brain injury, including white matter lesions, microbleeds, infarcts, and dilated perivascular spaces, is an important mechanism underlying the relationship between hypertension and dementia^11^.

In subtype analyses, the effects of hypertension were consistent with the risks of vascular and Alzheimer’s dementia. Although the association of high BP with vascular dementia was pronounced in previous studies, the association with Alzheimer’s dementia is inconsistent^21–23^. However, compelling evidence shows that hypertension is associated not only with vascular dysfunction and cerebrovascular disease, but also with beta amyloid deposition, which is an emerging marker for risk of Alzheimer’s dementia^24–27^.

There is a lack of definitive support from RCTs regarding the effectiveness of controlling hypertension for prevention of dementia^8–11,26,28^. If hypertension is a risk factor for dementia only when present in midlife, as shown in this and previous studies, then preventing dementia by lowering BP would be effective only when initiated before 70 years of age. However, an RCT of BP intervention in these age groups would be very difficult because a large sample and long follow-up would be required due to the fact that most cases of incident dementia occur in later life^11^. In several observational and meta-analysis studies, lowering SBP has been suggested to reduce the risk of cognitive impairment, and the evidence is stronger for lowering BP in middle age compared with later in life^3,8,29^. However, most observational studies regarding BP effects on dementia risk in midlife used a single BP measurement and provided no information regarding hypertension control between this measurement and the onset of dementia. Therefore, in the present study, the effects of BP changes were investigated, and age-specific optimal BP levels were determined using follow-up BP measurements.

Our findings of a log-linear association of hypertension burden with dementia suggest cumulative effects of hypertension on cognitive function. These are consistent with the results of previous studies that have suggested that sustained hypertension in mid- and late-life is associated with higher risk of subsequent dementia^30,31^. Associations between hypertension burden and each dementia subtype have not been previously reported. In this study, vascular dementia risk was more strongly affected by total cumulative exposure to hypertension than was Alzheimer risk, suggesting adequate BP control might help minimize the risk of dementia, especially in patients with more vascular risk factors.

Previous studies showed that low SBP is related to risk of dementia^32,33^. Low BP could result in decreased blood flow to the brain. In this study, a U-shaped relationship between delta SBP and the risk of dementia was observed. This result indicated that both excessively low and excessively high BP may fail to prevent dementia. Among individuals < 70 years of age, SBP of 120–139 mmHg was the optimal range for reducing risk of dementia. Although patients with baseline SBP < 120 mmHg did not have significantly increased risk of dementia compared with those with baseline SBP 120 ~ 139 mmHg, large positive and negative changes of SBP (delta SBP) were associated the increased risk of dementia in subjects < 70 years of age (presented in Fig. 6). The results indicate the appropriate target BP ranges for future trials examining reduction of BP for dementia prevention.

### Limitations

This study has several limitations. First, this retrospective and non-randomized study cannot prove or disprove causal relationships. Studies using administrative databases might be susceptible to errors arising from coding inaccuracies. To minimize this problem, the definition previously validated in studies that used the Korean NHIS sample cohort was applied^16–18,34^. Second, although the repeated measurements of initially elevated BP used in this study could reduce the risk of overestimation through an association with a median 8 mmHg decrease in SBP (compared with initial readings)^35^, BP was measured at a single visit. Uniformity of BP measuring devices was lacking since health examinations were performed in different hospitals and clinics. Nevertheless, instruments for BP measurements in all health examination institutions receive quality assessment every 3 years according to the Basic Act on National Health Examination^15,16,18^. Third, this study used the measurement of BP only at the follow-up timepoints. The definition of hypertension burden assumes that the BP was elevated on all days between the measurements and this does not have to be true. Fourth, although patients with diagnosed dementia were excluded, baseline cognitive function was not assessed; thus, the influence of mild baseline cognitive dysfunction could not be excluded or examined. Last, only Asians were enrolled in the present study, and it is unclear whether the results can be extrapolated to other populations. Despite these limitations, this study presents the largest population dataset in the literature used to investigate the association between optimal BP control and dementia outcome in the general population.

## Conclusion

Increased SBP and DBP were associated with higher risks of dementia, although these effects might be attenuated in older individuals. Cumulative hypertension burden was log-linearly associated with dementia risk, and adequate BP control was associated with lower risk of dementia in subjects < 70 years of age, suggesting that reducing the burden might help to minimize the risk of subsequent dementia in middle-aged patients.

## Supplementary Information

Supplementary Information.
