# Supplementary material for: Associations of hypertension burden on subsequent dementia: a population-based cohort study
Source: Sci Rep. 2021 Jun 10;11:12291. doi: 10.1038/s41598-021-91923-8 (PMC8192762; doi:10.1038/s41598-021-91923-8)
Supplement: Supplementary file 1 — Supplementary Information. [file 41598_2021_91923_MOESM1_ESM.docx]

**Supplementary Materials**

***Associations of hypertension burden on subsequent dementia:***

***A population-based cohort study***

**Supplementary Methods**

***BP measurement***

BP measurements were performed at local hospitals and clinics certified as medical health examination centers by the Korean National Health Insurance Corporation.^1-3^ After ≥5 minutes of rest in the sitting position, BP was measured at the brachial artery by qualified medical personnel. Automatic oscillometric devices or mercury sphygmomanometers were used, with the choice of device left to the discretion of individual examination centers. If the first BP measurement was >120/80 mmHg, the measurement was repeated once to prevent overestimation.^2, 3^ The main analysis used BP measured at the index date (initial health screening).

***Comorbidities and outcomes***

Use of prescription medication was ascertained by identifying database claims within 90 days before the index date. Income status was evaluated based on total amount of national health insurance premiums paid by the insured individual in the index year, which is proportional to the individual’s income.

Diagnosis of dementia was defined using the following ICD-10 codes of the Korean government for covering medical expenditure for patients with dementia: F00 and G30 for Alzheimer’s disease, F01 for vascular dementia, F02 for dementia with other diseases classified elsewhere, and F03 for unspecified dementia. To evaluate the accuracy of our definition of dementia, a validation study was conducted in two teaching hospitals with a total of 972 patients. Patient medical records and results of cognitive function tests including the Mini Mental State Examination were reviewed by three physicians (HJ, ISK, and PSY). The positive predictive value was 94.7% (920/972).

***Statistical analysis***

Descriptive statistics were used to characterize baseline characteristics and comorbidities. Continuous variables were expressed as the mean ± standard deviation (SD), and categorical variables were reported as frequency (percentage). Incidence rates of dementia were calculated by dividing the number of events by person-time at risk, with the 95% confidence interval (CI) estimated by exact Poisson distribution.

All tests were two-tailed, with P-value <0.05 considered statistically significant. Statistical analyses were conducted using SAS version 9.3 (SAS Institute, Cary, NC, USA), SPSS version 23.0 statistical package (SPSS Inc., Chicago, IL, USA), and R statistical software, version 3.5.3 (R Foundation for Statistical Computing, Vienna, Austria).

**References for Supplementary Methods**

1. Son JS, Choi S, Kim K*, et al.* Association of Blood Pressure Classification in Korean Young Adults According to the 2017 American College of Cardiology/American Heart Association Guidelines With Subsequent Cardiovascular Disease Events. *Jama* 2018;**320**:1783-92.

2. Kim TH, Yang PS, Yu HT*, et al.* Effect of hypertension duration and blood pressure level on ischaemic stroke risk in atrial fibrillation: nationwide data covering the entire Korean population. *Eur Heart J* 2019;**40**:809-19.

3. Kim D, Yang PS, Kim TH*, et al.* Ideal Blood Pressure in Patients With Atrial Fibrillation. *J Am Coll Cardiol* 2018;**72**:1233-45.

**Supplementary Table 1.** Definitions and ICD-10 codes used for defining comorbidities and outcome.

| **Comorbidities** | **Definitions** | **ICD-10 codes or conditions** |
| --- | --- | --- |
| Heart failure | Defined from diagnosis* | I11.0, I50, I97.1 |
| Diabetes mellitus | Defined from diagnosis* plus treatment | E10, E11, E12, E13, E14  Treatment: all kinds of oral antidiabetics and insulin. |
| Hypertension | Defined from diagnosis* | I10, I11, I12, I13, I15 |
| Dyslipidemia | Defined from diagnosis* | E78 |
| Prior ischemic stroke | Defined from diagnosis* | ICD10: I63, I64 |
| Prior transient ischemic attack | Defined from diagnosis* | G45 |
| Prior hemorrhagic stroke | Defined from diagnosis* | I60, I61, I62 |
| Prior myocardial infarction | Defined from diagnosis* | I21, I22, I25.2 |
| Peripheral arterial disease | Defined from diagnosis* | I70.0, I70.1, I70.2, I70.8, I70.9 |
| Chronic obstructive pulmonary disease | Defined from diagnosis* plus treatment | J42, J43(except J43.0), J44  Treatment: SABA, SAMA, LABA, LAMA, ICS, ICS+LABA, or methylxanthine (>1 month). |
| Chronic kidney disease | Defined from eGFR (if laboratory value was not available, diagnosis* was used) | eGFR <60 mL/min per 1.73 m^2^  N18, N19 |
| Liver disease | Defined from diagnosis* of chronic liver disease, cirrhosis, and hepatitis | B18, K70, K71, K72, K73, K74, K76.1 |
| Malignant neoplasm | Defined from diagnosis* | C00-C97 |
| Household income status | Categorized into three groups, based on the total amount of national health insurance premiums paid by an insured person in each year which is proportional to the person’s income | High: subjects with upper 30% income  Middle: subjects between high and low  Low: subjects with lower 40% income or under the Medical Aid program |
| **Clinical outcomes** | **Definitions** | **ICD-10 codes or conditions** |
| Dementia | Defined from diagnosis with prescription of dementia drugs | F00, G30, F01, F02, F03, G31  Dementia drugs: rivastigmine, galantamine, memantine, or donepezil |

*To ensure accuracy, comorbidities were established based on one inpatient or two outpatient records of ICD-10 codes in the database.

eGFR, estimated glomerular filtration rate.

**Supplementary Table 2.** Baseline characteristics of patients according to age subgroups.

| **Variables** | **Age 40-59 years**  **(n=292,822)** | **Age 60-69 years**  **(n=96,306)** | **Age 70-79 years**  **(n=39,848)** | **p value** |
| --- | --- | --- | --- | --- |
| Dementia event | 1,023 | 3,518 | 4,894 | <0.001 |
| Vascular dementia | 213 | 471 | 579 | <0.001 |
| Alzheimer dementia | 649 | 2,586 | 3,691 | <0.001 |
| Age, years | 50.4 ± 4.5 | 63.9 ± 2.8 | 72.9 ± 2.7 | <0.001 |
| Male | 165,685 (56.6) | 48,930 (50.8) | 18,765 (47.1) | <0.001 |
| Initial SBP, mmHg | 123.6 ± 15.7 | 129.7 ± 17.1 | 132.5 ± 17.9 | 0.003 |
| Initial DBP, mmHg | 78.0 ± 10.7 | 79.4 ± 10.7 | 79.3 ± 10.8 | 0.050 |
| Hypertension at baseline | 60,623 (20.7) | 43,098 (44.8) | 22,804 (57.2) | <0.001 |
| Hypertension duration at baseline, months | 2.9 ± 1.7 | 3.3 ± 1.7 | 3.4 ± 1.7 | <0.001 |
| High tertile of income | 168,557 (57.6) | 47,010 (48.8) | 21,476 (53.9) | <0.001 |
| Body mass index, kg/m^2^ | 24.0 (2.8) | 24.1 (3.0) | 23.5 (3.2) | <0.001 |
| Alcohol consumption, times/week |  |  |  | <0.001 |
| <1 | 192,036 (70.7) | 67,105 (77.8) | 29,065 (81.7) |  |
| 1-2 | 52,244 (19.2) | 9,690 (11.2) | 2,735 (7.7) |  |
| 3-4 | 19,083 (7.0) | 4,936 (5.7) | 1,504 (4.2) |  |
| ≥5 | 8,216 (3.0) | 4,550 (5.3) | 2,264 (6.4) |  |
| Missing values | 21,243 | 10,025 | 4,280 |  |
| Smoking, No. (%) |  |  |  | <0.001 |
| Never | 190,264 (68.6) | 70,434 (77.4) | 30,116 (80.2) |  |
| Ex | 27,478 (9.9) | 7,329 (8.0) | 2,900 (7.7) |  |
| Current | 59,704 (21.5) | 13,291 (14.6) | 4,529 (12.1) |  |
| Missing values | 15,376 | 5,252 | 2,303 |  |
| Physical activity, times/week |  |  |  | <0.001 |
| 0-2 | 208,319 (77.2) | 63,959 (74.7) | 27,778 (78.8) |  |
| 3-4 | 37,125 (13.8) | 9,115 (10.7) | 2,403 (6.8) |  |
| ≥5 | 24,281 (9.0) | 12,508 (14.6) | 5,053 (14.3) |  |
| Missing values | 23,097 | 10,724 | 4,614 |  |
| Hypothyroidism | 7,070 (2.4) | 2,688 (2.8) | 981 (2.5) | 0.004 |
| Hyperthyroidism | 6,923 (2.4) | 2,710 (2.8) | 1,037 (2.6) | <0.001 |
| Major bleeding | 2,018 (0.7) | 1,225 (1.3) | 774 (1.9) | <0.001 |
| Atrial fibrillation | 4,677 (1.6) | 3,754 (4.0) | 2,599 (6.7) | <0.001 |
| Heart failure | 4,243 (1.4) | 4,419 (4.6) | 3,638 (9.1) | <0.001 |
| Diabetes mellitus | 15,986 (5.5) | 11,494 (11.9) | 5,545 (13.9) | <0.001 |
| Dyslipidemia | 57,744 (19.7) | 30,631 (31.8) | 12,411 (31.1) | <0.001 |
| Prior Myocardial infarction | 1,642 (0.6) | 1,445 (1.5) | 956 (2.4) | <0.001 |
| Peripheral artery disease | 3,372 (1.2) | 2,827 (2.9) | 1,473 (3.7) | <0.001 |
| Chronic kidney disease | 1,541 (0.5) | 948 (1.0) | 468 (1.2) | <0.001 |
| COPD | 16,072 (5.5) | 13,840 (14.4) | 9,528 (23.9) | <0.001 |
| Liver disease | 56,437 (19.3) | 23,004 (23.9) | 8,991 (22.6) | <0.001 |
| Malignant neoplasm | 14,716 (5.0) | 8,684 (9.0) | 4,904 (12.3) | <0.001 |
| Medication use at baseline |  |  |  |  |
| Aspirin | 21,314 (7.3) | 18,337 (19.0) | 9,909 (24.9) | <0.001 |
| Statin | 21,296 (7.3) | 13,515 (14.0) | 5,334 (13.4) | <0.001 |
| Oral anticoagulant | 519 (0.2) | 450 (0.5) | 286 (0.7) | <0.001 |
| ß-blocker | 24,587 (8.4) | 17,197 (17.9) | 9,115 (22.9) | <0.001 |
| RAAS blocker | 25,061 (8.6) | 17,733 (18.4) | 9,231 (23.2) | <0.001 |
| Calcium channel blocker | 30,297 (10.3) | 23,304 (24.2) | 12,523 (31.4) | <0.001 |
| Diuretics | 26,804 (9.2) | 21,261 (22.1) | 12,495 (31.4) | <0.001 |
| Alpha blocker | 6,008 (2.1) | 5,443 (5.7) | 3,357 (8.4) | <0.001 |

Continuous variables were expressed as mean ± standard deviation, and categorical variables were expressed as counts and percentages. Baseline characteristics were compared across the three groups using the Chi-square test or Fisher’s exact test for categorical variables and the ANOVA or Kruskal-Wallis test for continuous variables. COPD, chronic obstructive pulmonary disease; DBP, diastolic blood pressure; RAAS, renin-angiotensin-aldosterone system; SBP, systolic blood pressure.

**Supplementary Table 3.** The number and incidence rates of overall, vascular and Alzheimer’s dementia according to age subgroup.

| **Variables** | **Age 40-59 years (n=292,822)** | **Age 60-69 years**  **(n=96,306)** | **Age 70-79 years**  **(n=39,848)** | **p value** |
| --- | --- | --- | --- | --- |
| Overall dementia |  |  |  |  |
| Event, n | 1,023 | 3,518 | 4,894 |  |
| Person-years | 292,822 | 96,306 | 39,848 |  |
| Incidence (/100 person-years) | 0.05 (0.04-0.05) | 0.51 (0.50-0.53) | 1.88 (1.82-1.93) | <0.001 |
| Vascular dementia |  |  |  |  |
| Event, n | 213 | 471 | 579 |  |
| Person-years | 292,822 | 96,306 | 39,848 |  |
| Incidence (/100 person-years) | 0.01 (0.01-0.01) | 0.07 (0.06-0.07) | 0.21 (0.20-0.23) | <0.001 |
| Alzheimer’s dementia |  |  |  |  |
| Event, n | 649 | 2,586 | 3,691 |  |
| Person-years | 292,822 | 96,306 | 39,848 |  |
| Incidence (/100 person-years) | 0.03 (0.03-0.03) | 0.38 (0.36-0.39) | 1.40 (1.36-1.45) | <0.001 |

Incidence rate was calculated by dividing the number of events by person-time at risk, with the 95% confidence interval estimated by exact Poisson distribution. Exact test was used in comparing two incidence rates.

**Supplementary Figure 1**. Cumulative hazard and event of dementia according to the time in different systolic blood pressure (SBP) group.


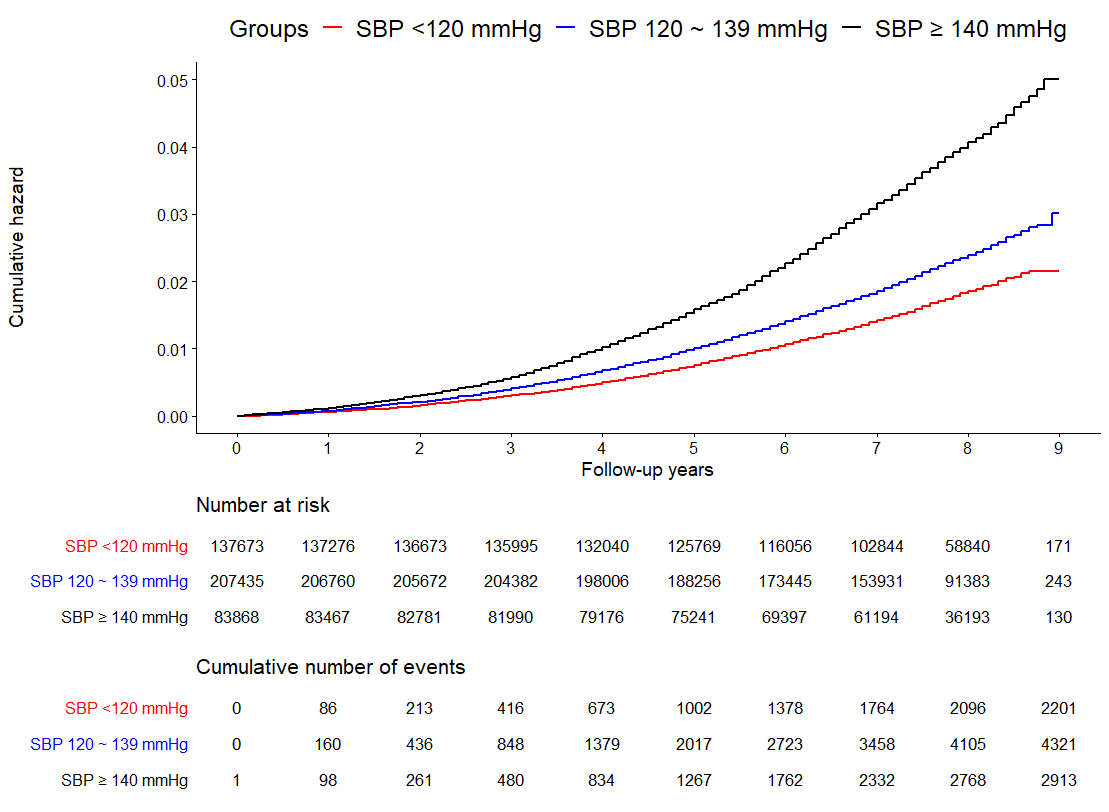


**Supplementary Figure 2**. Boxplots for distribution of baseline systolic blood pressure (left panel) and diastolic blood pressure (left panel) according to age subgroups. The box represents interquartile range (IQR; between the 25th and 75th percentiles). Upper and lower whiskers represent 75th percentile (Q3) plus 1.5 times IQR and the 25th percentile (Q1) minus 1.5 times IQR, respectively.


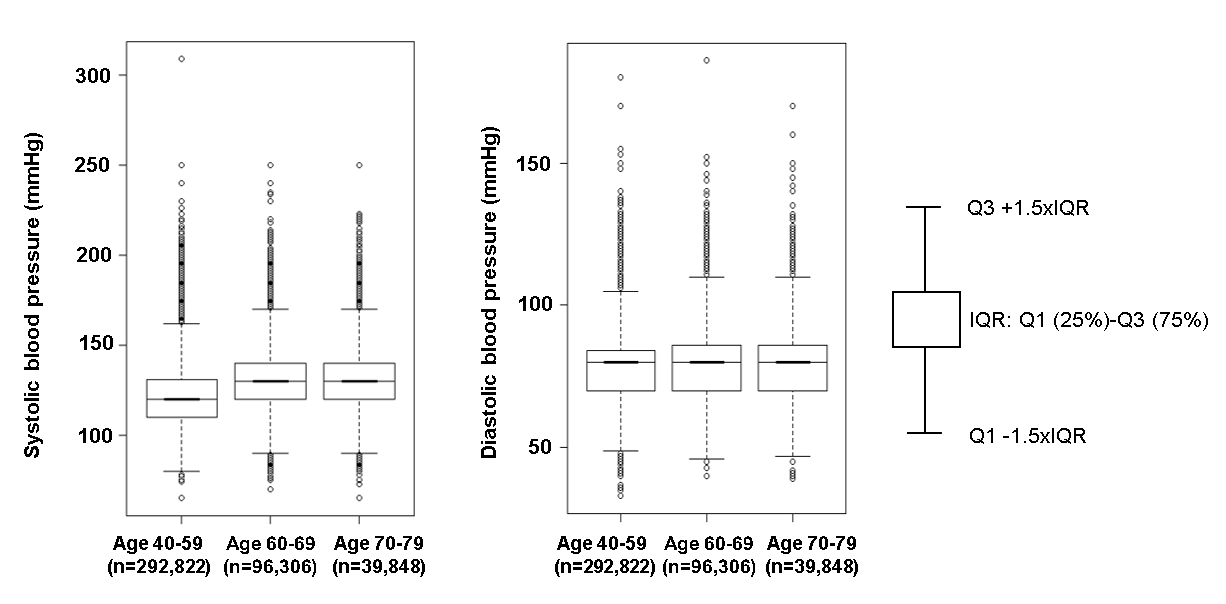


**Supplementary Figure 3**. Adjusted HRs of risk of dementia according to baseline SBP (A) and DBP (B) in subjects including those with a previous history of ischemic stroke / transient ischemic attack (n=22,694) or hemorrhagic stroke (n=1,140).


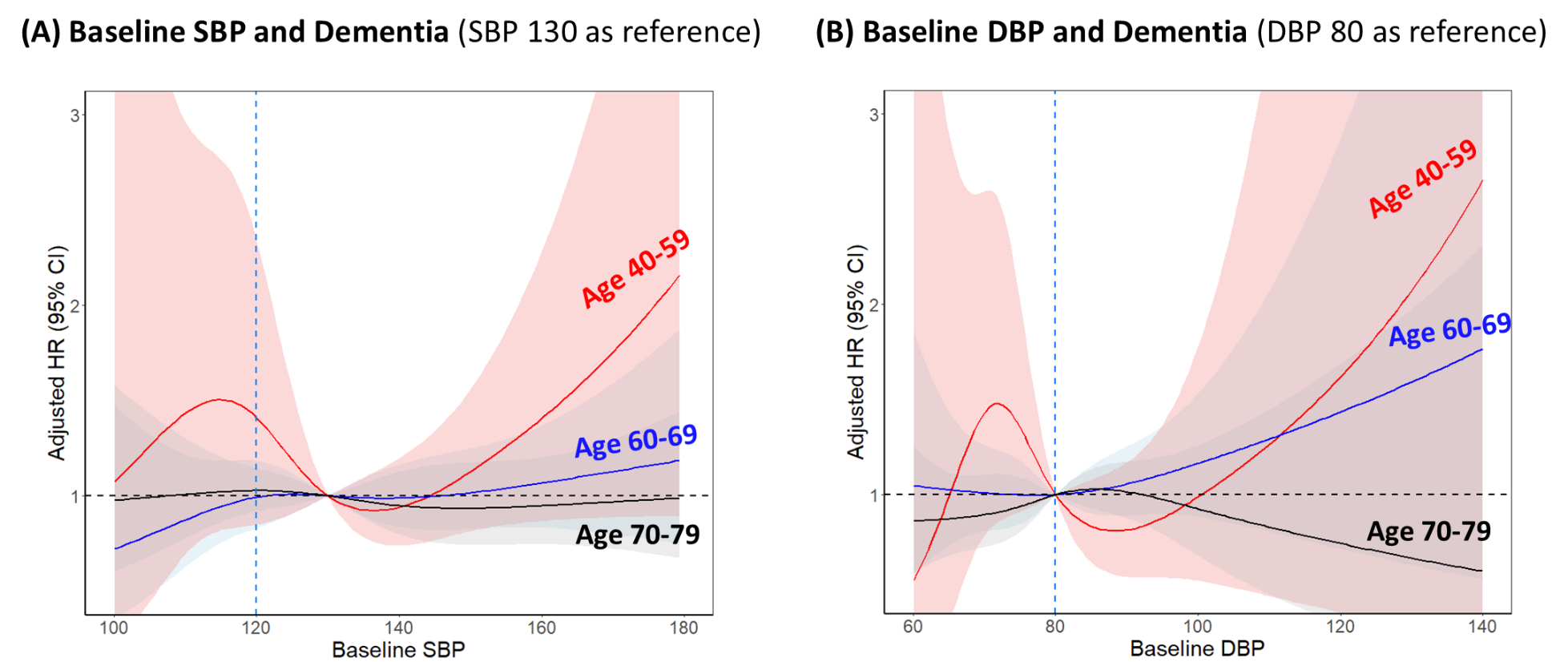


CI, confidence interval; DBP, diastolic blood pressure; HR, hazard ratio; SBP, systolic blood pressure.

**Supplementary Figure 4**. Baseline SBP and adjusted HRs for risk of dementia after censoring for incident ischemic stroke measured in different age subgroups. Patients with baseline SBP 120mmHg are used as reference.


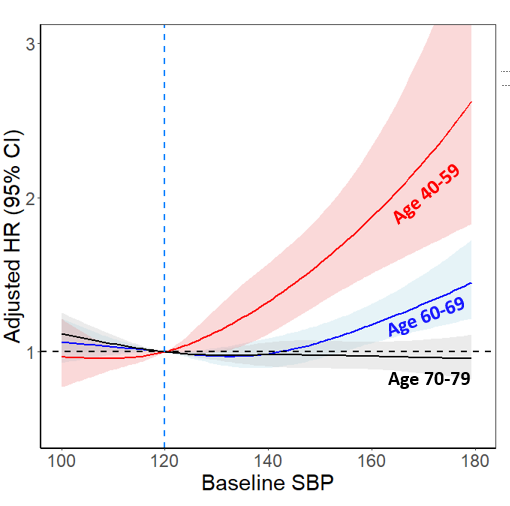


CI, confidence interval; HR, hazard ratio; SBP, systolic blood pressure.

**Supplementary Figure 5**. Risk of dementia according to baseline SBP categories (<120, 120–139 [reference], ≥140 mmHg) in different age subgroups.


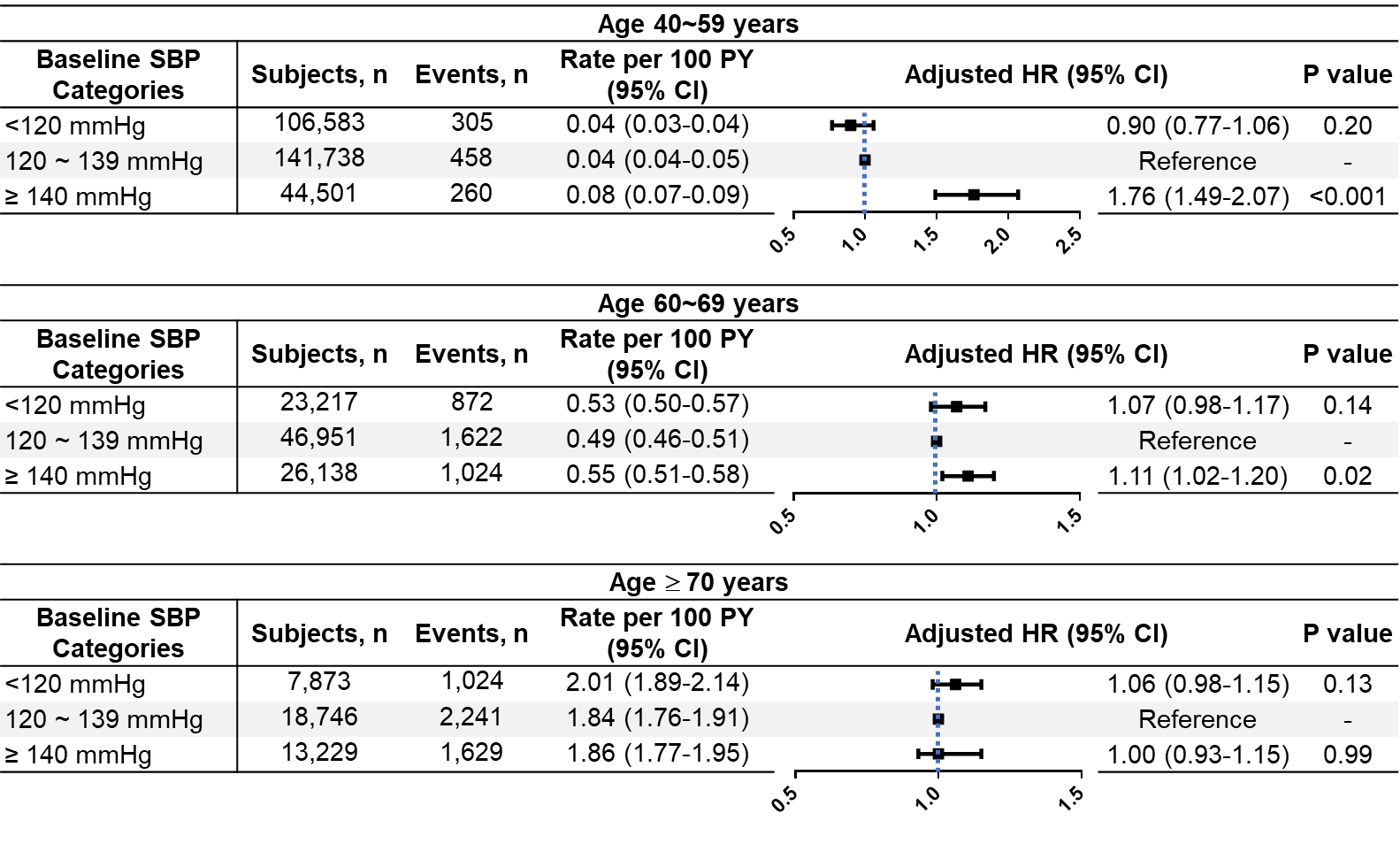


CI, confidence interval; HR, hazard ratio; SBP, systolic blood pressure; PY, person-years.

**Supplementary Figure 6.** Risk of dementia according to baseline DBP categories (<80 [reference], 80-89, ≥90 mmHg) in different age subgroups.


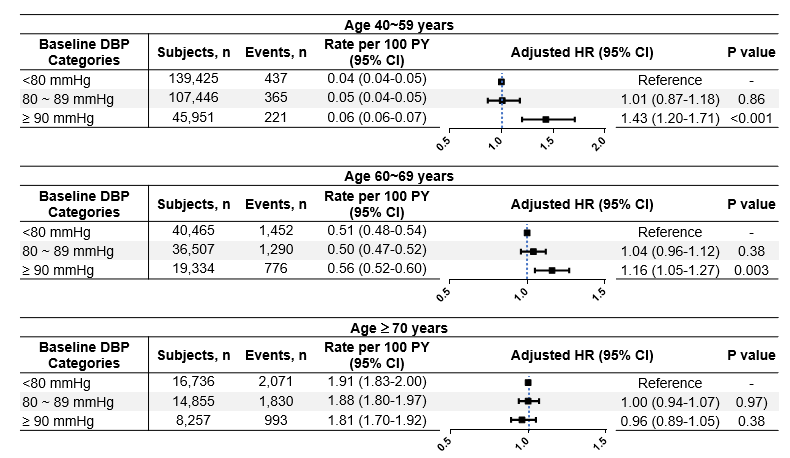


CI, confidence interval; DBP, diastolic blood pressure; HR, hazard ratio; PY, person-years.

**Supplementary Figure 7.** An analysis of effects of different classes of antihypertensive medications at baseline on subsequent dementia risk.


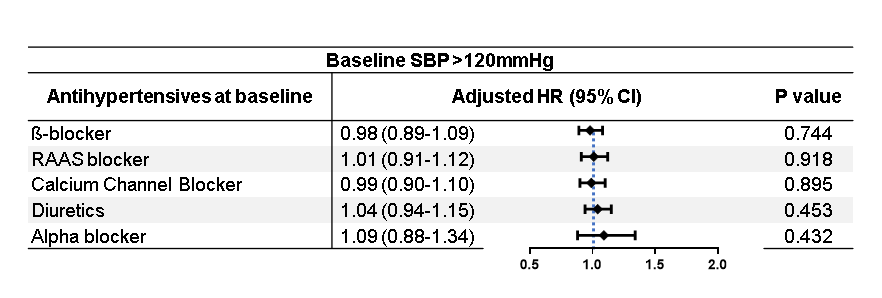


A multivariate Cox proportional hazards regression model was used to analyze the effects of different classes of antihypertensive medications at baseline on subsequent dementia risk. The model was adjusted by age, sex, income, body mass index, alcohol consumption, smoking, physical activity, hypothyroidism, hyperthyroidism, major bleeding, atrial fibrillation, heart failure, diabetes mellitus, dyslipidemia, myocardial infarction, peripheral artery disease, chronic kidney disease, chronic obstructive pulmonary disease, liver disease, malignant neoplasm, and medication at baseline.

CI, confidence interval; HR, hazard ratio; RAAS, renin-angiotensin-aldosterone system; SBP, systolic blood pressure.
